# Sprue

**Published:** 1917-01

**Authors:** 


**Sprue.** Thos. R. Brown, Baltimore, Johns Hopkins Hosp. Bull., Oct., quotes Bahr's opinion that sprue is a specific disease, especially prevalent in Ceylon, affecting Europeans especially but not exclusively, variable in severity, with absence or marked failure of intestinal ferments, probably an alimentary toxaemia and probably due to the thrush fungus, monilia albicans. Attempts to convey the disease to lower animals, even monkeys, have failed, though Ashford claims to have identified the monilia from over 100 cases as a distinct, pathogenic species, which produces a fatal mycotic septicaemia in certain laboratory animals. The author's case showed achlorhydria, much diminished peptic power, and failure of amylase, trypsin and lipase of the pancreas.

